# Association between Sacral Fractures and Pelvic Ring Injuries: A Correlative Study of Fracture Morphologies

**DOI:** 10.1055/s-0046-1820458

**Published:** 2026-06-16

**Authors:** João Victor Jordão Sousa, João Pedro Lemos Soares, Antenor Aguiar Almeida Junior, Tomas Costa Arslanian, Eriko Gonçalves Filgueira

**Affiliations:** 1 Orthopedic and Traumatology Service, Hospital de Base do Distrito Federal, Brasília, DF, Brazil

**Keywords:** classification, pelvic bones, sacrum, classificação, ossos pélvicos, sacro

## Abstract

**Objective:**

To investigate, in a retrospective cohort, the association between sacral fractures and pelvic ring injuries (PRIs) by correlating their respective classification subtypes.

**Methods:**

We selected 45 patients admitted to a tertiary trauma center between March 2023 and May 2025. Imaging studies were evaluated for the presence of PRIs and sacral fractures, followed by their classification according to the Tile and Denis systems. Then, a statistical analysis was performed using the McNemar–Bowker test and the Cramér's V coefficient to assess the strength of the association.

**Results:**

Among the PRIs classified according to the Tile system, type B was predominant (62.22%), followed by type C (35.56%), and type A (2.22%). Among the sacral fractures classified according to the Denis system, type I was the most prevalent (50%), followed by type II (28.57%), and type III (21.43%). When the classifications were correlated, Tile type-B PRIs showed an 85.7% incidence of associated sacral fractures, 66.6% of which were Denis type I. For Tile type-C PRIs, the incidence of associated sacral fractures was of 100%, with 50% classified as Denis type II. There was a significant association between the Tile and Denis classifications ( *p* < 0.001), with a moderate correlation (Cramér's V = 0.27–0.35).

**Conclusion:**

The study suggests correlations between PRIs and sacral fracture subtypes, with Tile B-Denis I and Tile C-Denis II representing the most frequent associations. This relationship may facilitate diagnostic investigation and improve patient outcomes.

## Resumo

**Objetivo:**

Investigar, por meio de coorte histórica, a associação entre fraturas sacrais e lesões do anel pélvico (LAPs), por meio da correlação entre seus subtipos classificatórios.

**Métodos:**

Foram selecionados 45 pacientes internados em um centro traumatológico terciário entre março de 2023 e maio de 2025. Foram avaliados exames de imagem quanto à presença de LAPs e fraturas sacrais. Em seguida, os pacientes foram classificados segundo as classificações de Tile e de Denis. Então, foram submetidos à análise estatística seguindo a metodologia do teste de McNemar–Bowker e para avaliar a força da associação por meio do coeficiente V de Cramér.

**Resultados:**

Na classificação das LAPs por Tile, encontrou-se predominância do tipo B (62,22%), seguido do tipo C (35,56%) e do tipo A (2,22%). Quanto às fraturas sacrais classificadas por Denis, o tipo mais prevalente foi I (50%), seguido do tipo II (28,57%) e do tipo III (21,43%). Ao associar os dados, observou-se que nas LAPs Tile B houve uma incidência de 85,7% de fratura sacral associada, sendo que, destas, 66,6% eram Denis I. Já nas LAPs Tile C, a incidência de fratura sacral associada foi de 100%, sendo 50% descritas como Denis II. Houve associação significativa entre as classificações de Tile e de Denis ( *p* < 0,001), com correlação moderada (V de Cramér: 0,27–0,35).

**Conclusão:**

O estudo sugere correlações entre subtipos de LAP e fraturas sacrais, sendo mais frequentes as associações Tile B-Denis I e Tile C-Denis II. Essa associação pode acelerar a investigação e melhorar os desfechos dos pacientes.

## Introduction

Pelvic ring injuries (PRIs) and their variants have been the subject of academic debate for over a century, from Malgaigne's descriptions in 1859 1 to more recent systematic reviews. 2 3

These injuries encompass a broad spectrum and present several classifications and subtypes; PRIs occur across all age groups and account for approximately 3% of all fractures. They are more common in male subjects aged 18 to 44 years. 4 Traumatic disruptions of the pelvic ring are most commonly the result of high-energy trauma mechanisms and represent an important cause of morbidity and mortality. 5 6 7 In the literature, 2 8 mortality rates range from 10 to 40%, depending on the trauma mechanism, the previous clinical status of the patient, and the therapeutic management instituted.

Sacral fractures are relatively rare injuries. However, in PRIs, it is estimated that 10 to 45% of the cases are associated with such fractures, 9 10 with up to 30% of the cases not diagnosed in the first evaluation, resulting in poor outcomes for these patients. 11 12

Several classification systems have been developed to better understand and characterize this group of injuries. Among them, the most widely used are the Tile classification, which addresses pelvic ring stability, 13 and the Denis classification, which describes the morphology of sacral fractures. 4

Some studies have addressed the association between sacral fractures and PRIS. However, the literature remains scarce regarding correlations involving their respective subtypes in an attempt to determine the incidence of these synchronous injuries. 14 15 16

The objective of the current study was to investigate, through a retrospective evaluation, the association between sacral fractures and PRIs by correlating their respective subtypes.

## Materials and Methods

We conducted a retrospective cohort evaluation through a comprehensive review of the database of a tertiary trauma referral hospital to identify PRIs and sacral fractures between March 2023 and May 2025. The evaluation identified 55 cases of PRIs, sacral fractures, and both in patients who required hospitalization.

The inclusion criteria were the presence of PRIs, with or without associated sacral fractures, of traumatic etiology, as well as the availability of radiographic (X-ray) and computed tomography (CT) examinations for injury classification. The exclusion criteria were patients with incomplete complementary studies, non-traumatic etiology, or skeletal immaturity.

After screening, 10 patients were excluded from the study: 1, due to skeletal immaturity, 5, due to incomplete complementary examinations, and 4, because their imaging studies were not available in the radiological archive. As such, 45 participants were eligible for analysis.

**Fig. 1:**
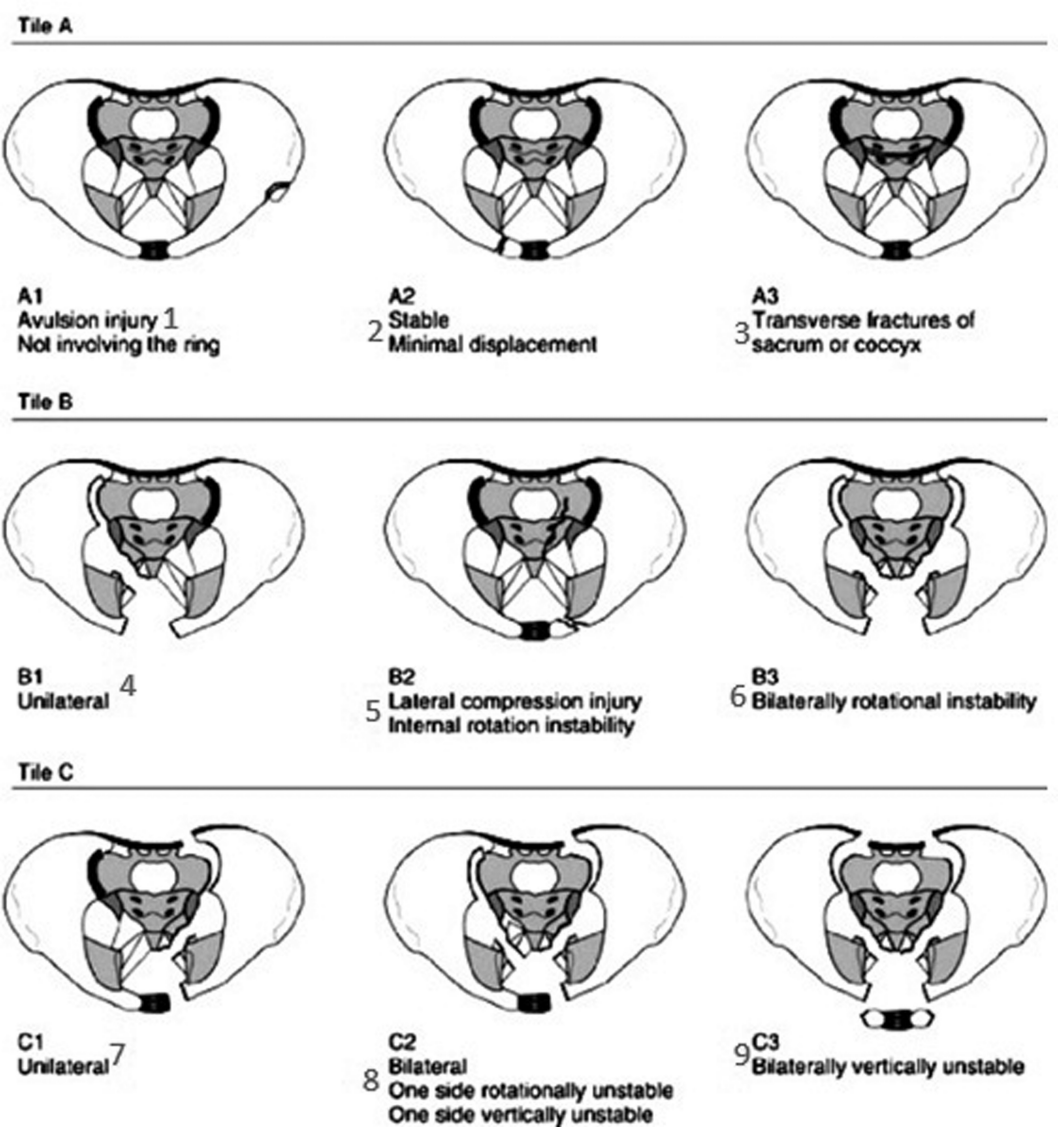
Tile classification of pelvic ring injuries.

**Fig. 2:**
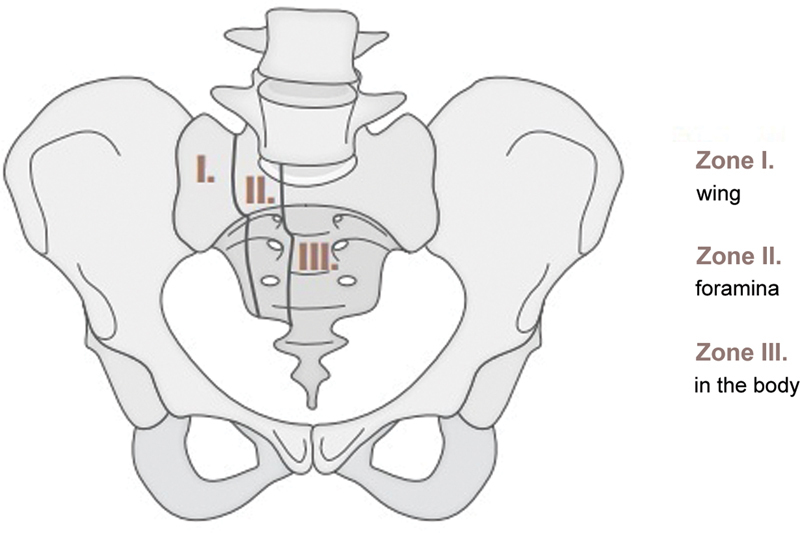
Denis classification of sacral fractures.

Following patient selection, digital pelvic radiographs were retrieved in anteroposterior, inlet, and outlet views, as well as CT scans with reconstructions in axial, sagittal, and coronal planes, all performed in a standardized manner at the same radiology center. The PRIs were subsequently evaluated and classified according to the Tile algorithm 13 ( Fig. 1 ), and the sacral fractures, according to the Denis classification ( Fig. 2 ), 11 by four independent examiners (JPLS, AAAJ, JVJS, TCA—resident physicians of the Orthopedic Trauma Unit). In cases of disagreement, a fifth senior examiner (EGF—orthopedic trauma and spine surgeon with 25 years of experience) reassessed the classification.

The data were stored in the Research Electronic Data Capture (REDcap, Vanderbilt University) application, maximizing data security and facilitating subsequent statistical analysis.

A descriptive analysis of the data presented measures of central tendency and dispersion for the numerical variables, as well as absolute and relative frequencies for the categorical variables (Tile and Denis classifications). The McNemar–Bowker test evaluated the associations involving these variables, with paired data arranged in a 3 × 3 contingency table and subsequently submitted to the Bowker function of the ANSM5 package (version 1.1.1) in the R (R Foundation for Statistical Computing) software. Cramér's V coefficient quantified the strength of the association. The institutional Research Ethics Committee, affiliated with Plataforma Brasil, approved the current study under CAAE number 85686924.9.0000.5553.

## Results

A total of 45 patients were evaluated regarding their radiographic and tomographic presentation of PRIs and sacral fractures ( Table 1 ).

**Table 1:** Clinical cases

Case	Age (years)	Gender	Trauma mechanism	Treatment center	Tile classification (X-ray)	Tile classification (CT)	Denis classification (X-ray)	Denis classification (CT)
**1**	67	Female	Pedestrian run-over	HBDF	C	B	2	2
**2**	74	Female	Pedestrian run-over	HBDF	C	C	2	1
**3**	47	Male	Pedestrian run-over	HBDF	C	C	2	2
**4**	22	Male	Motorcycle fall	HBDF	B	B	1	1
**5**	23	Female	Motorcycle fall	HBDF	B	B	1	1
**6**	54	Male	Pedestrian run-over	HBDF	C	C	1	2
**7**	18	Male	Motorcycle fall	HBDF	C	C	1	2
**8**	56	Female	Pedestrian run-over	HBDF	B	B	1	1
**9**	26	Female	Fall from height	HBDF	C	C	2	3
**10**	46	Female	Pedestrian run-over	HBDF	A	B	Fracture not detected	1
**11**	40	Female	Pedestrian run-over	HBDF	B	C	2	2
**12**	27	Male	Fall from height	HBDF	A	A	1	1
**13**	56	Male	Motorcycle fall	HBDF	C	C	1	2
**14**	34	Male	Fall from height	HBDF	C	C	2	3
**15**	61	Male	Fall from height	HBDF	C	C	2	3
**16**	NA	Male	Motor vehicle accident	HBDF	C	C	2	2
**17**	39	Male	Motorcycle fall	HBDF	B	B	2	2
**18**	56	Male	Motorcycle fall	HBDF	B	B	2	3
**19**	37	Male	Pedestrian run-over	HBDF	B	B	1	1
**20**	50	Male	Motorcycle fall	HBDF	C	C	Fracture not detected	3
**21**	20	Male	Pedestrian run-over	HBDF	B	B	1	2
**22**	44	Male	Motorcycle fall	HBDF	B	B	Fracture not detected	3
**23**	32	Male	Pedestrian run-over	HBDF	B	B	2	1
**24**	23	Male	Fall from height	HBDF	B	B	2	1
**25**	84	Male	Pedestrian run-over	HBDF	B	B	1	1
**26**	58	Male	Motorcycle fall	HBDF	B	B	1	3
**27**	23	Female	Pedestrian run-over	HBDF	C	C	2	1
**28**	23	Male	Pedestrian run-over	HBDF	C	C	1	2
**29**	32	Male	Motor vehicle accident	HBDF	B	Fracture not detected	1	1
**30**	61	Female	Fall from height	HBDF	B	B	1	1
**31**	82	Male	Pedestrian run-over	HBDF	B	B	Fracture not detected	Fracture not detected
**32**	66	Male	Fall from height	HBDF	B	B	1	1
**33**	58	Male	Motor vehicle accident	HBDF	B	B	3	3
**34**	63	Male	Fall from height	HBDF	B	B	1	1
**35**	44	Female	Motor vehicle accident	HBDF	B	C	1	1
**36**	75	Male	Fall from height	HBDF	C	C	2	2
**37**	56	Male	Pedestrian run-over	HBDF	B	B	Fracture not detected	1
**38**	41	Male	Motorcycle fall	HBDF	B	B	1	1
**39**	35	Male	Motor vehicle accident	HBDF	B	B	1	Fracture not detected
**40**	50	Male	Fall from height	HBDF	B	B	1	1
**41**	19	Female	Motor vehicle accident	HBDF	B	B	2	2
**42**	49	Male	Pedestrian run-over	HBDF	B	B	1	1
**43**	22	Female	Motor vehicle accident	HBDF	B	B	Fracture not detected	Fracture not detected
**44**	19	Female	Pedestrian run-over	HBDF	B	B	2	1
**45**	26	Male	Fall from height	HBDF	C	C	3	3

Abbreviations: CT, computed tomography; HBDF, Hospital de Base do Distrito Federal, Brazil; X-ray, radiography.

The mean age of the participants was of 44 (range: 18–84) years. The sample consisted of 75.56% (n = 34) of male and 24.44% (n = 11) of female patients.

Regarding the trauma mechanism, pedestrian run-over was the most prevalent, accounting for 37.78% (n = 17), followed by falls from height (24.4%, n = 11), motorcycle falls (22.22%, n = 10), and motor vehicle accidents (15.56%, n = 7).

**Fig. 3:**
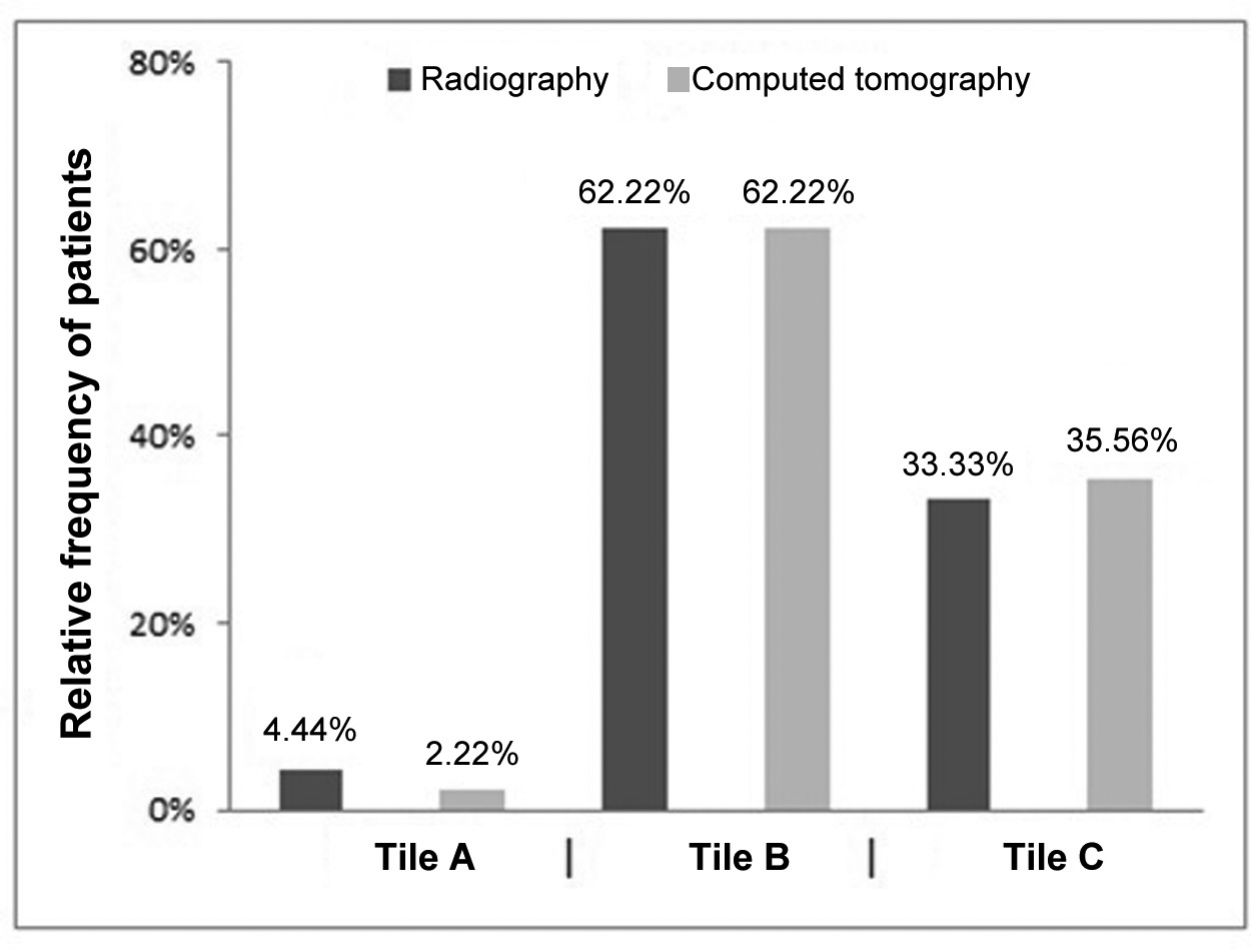
Radiographic and computed tomography evaluation of pelvic ring injuries.

For the PRIs, there were no statistically significant differences between radiographic and tomographic results. Tile type B was the most frequent (62.22%, n = 28), followed by type C (35.56%, n = 16), and type A (2.22%, n = 1) ( Fig. 3 ).

**Fig. 4:**
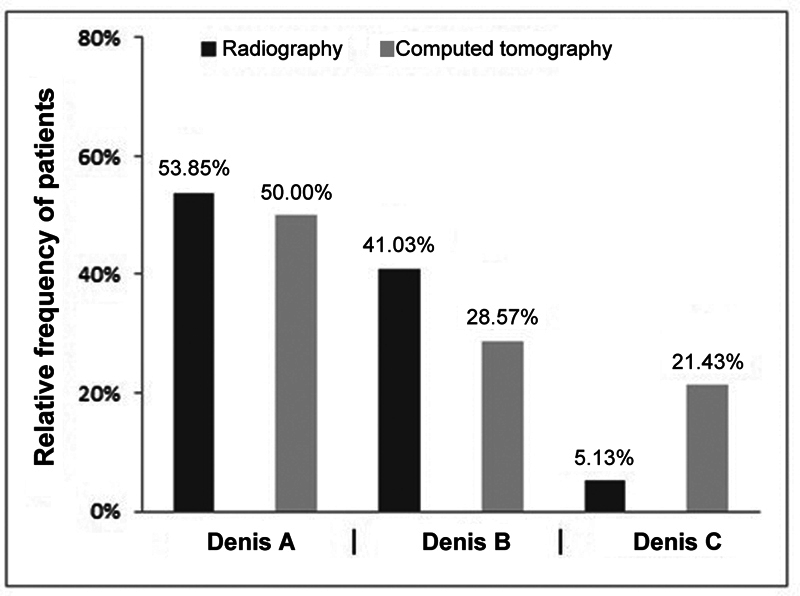
Radiographic and computed tomography evaluation of sacral fractures.

For sacral fractures, relevant differences were observed between the imaging methods. On the radiographic evaluation, Denis type I accounted for 53.85% of the cases (n = 21), followed by type II (41.03%, n = 16), and type III (5.13%, n = 2), totaling 39 evaluations. On the tomographic evaluation, type I accounted for 50.00% of the cases (n = 21), type II, for 28.57% (n = 12), and type III, for 21.43% (n = 9), totaling 42 evaluations ( Fig. 4 ). Sacral fractures were not identified in six radiographic evaluations; in three of these cases, they also remained undetected on the tomographic evaluation. These cases were excluded from the final statistical analysis for each imaging modality.

A statistically significant association between the Tile and Denis classifications was observed for CT scans and X-rays. For the CT scans, the McNemar–Bowker test showed χ ^2^_df = 3_ = 20.33, *p* < 0.001, with a moderate strength of association (Cramér's V = 0.35; 3 × 3 table). For the X-rays, the McNemar–Bowker test showed χ ^2^_df = 3_ = 26.4, *p* < 0.001, also with a moderate strength of association (Cramér's V = 0.27; 3 × 3 contingency table).

Among Tile type-B PRIs, the incidence of associated sacral fractures was of 85.7%, 66.6% of which were Denis type I. Among Tile type-C PRIs, the incidence of associated sacral fractures was of 100%, with 50% classified as Denis type II. Thus, in both imaging modalities, there was a relative concentration of Denis type-I fractures associated with Tile type-B injuries, and Denis type-II fractures associated with Tile type-C injuries ( Table 2 ).

**Table 2:** Contingency table

Radiography				
		***DENIS***		
		*1*	*2*	*3*
***TILE***	*A*	1	0	0
	*B*	16	7	1
	*C*	4	9	1
**McNemar–Bowker test**	χ ^2^ = 26.4	*p* < 0.001		
**Cramér's V**	0.279			
**Computed tomography**				
		***DENIS***		
		*1*	*2*	*3*
***TILE***	*A*	1	0	0
	*B*	16	4	4
	*C*	3	8	5
**McNemar–Bowker test**	χ ^2^ = 20.33	*p* < 0.001		
**Cramér's V**	0.352			

## Discussion

Pelvic ring injuries represent approximately 3% of traumatic skeletal injuries. Up to 80% of PRIs considered unstable result from high-energy trauma. 3 Sacral fractures are estimated to occur in 10 to 45% of these pelvic fractures. 9

In 1859, Malgaigne described the first recognized classification of PRIs, reporting the *double vertical fracture* . 1 However, the first comprehensive classification of these injuries was proposed only a century later by Tile. 13 who classified PRIs as stable (type-A fractures), rotationally-unstable (type-B), and vertically-unstable (type-C) 13 ( Fig. 1 ).

Sacral fractures are rare injuries and usually result from high-energy trauma. Their incidence remains debated in the literature 17 due to the difficulty in identifying these fractures during the acute phase. Among the reasons for this difficulty are limitations in imaging assessment caused by the overlap of soft tissues and intestinal gas on radiographs, as well as insufficient diagnostic suspicion, since patients often do not present evident neurological deficits during the initial evaluation. 16

Denis et al. 11 classified sacral fractures into three types according to their anatomical location ( Fig. 2 ): lateral to the foramina (zone I), foraminal (zone II), and medial to the foramina (zone III).

In the present study, the prevalence of PRIs was of 62% for Tile type B, 35% for Tile type C, and 2.2% for Tile type A, differing from the values originally reported by Tile. 13 Denis type-I fractures were the most prevalent (50%), followed by type II (28.6%) and type III (21.4%). Gänsslen et al. 18 observed 42% injuries to zone I, 47% to zone II, and 11% to zone III, differing from our results. However, studies 10 evaluating the relationship between trauma mechanism and sacral fracture patterns reported discrepant values for zone-II and zone-III injuries. These studies demonstrated that, in anteroposterior compression trauma, sacral fractures to zone III may account for up to 25% of the cases, which is consistent with the data herein presented. The current study identified a positive association between Tile type-B and Denis type-I fractures. Previous investigations have reported similar findings. Beckmann and Cai. 19 observed that, in vertically-stable subtypes, 48% of the lesions were to zone I, corroborating our results.

The present study also identified an association between Tile type-C and Denis type-II fractures. Previous studies 18 19 have reported a high incidence of zone-II and zone-III involvement in vertically-unstable injuries (Tile type C). Tötterman et al. 20 reported fractures to zone II in 72% and to zone III in 22% of the patients with this injury profile, which is consistent with our findings.

The current study has some limitations. As the sample consisted of patients who required admission to a tertiary hospital, there are epidemiological limitations resulting from the relatively-small number of eligible patients. Although statistical significance was achieved, the sample may reflect a tendency toward polytraumatized patients with complex cases and biomechanically-unstable pelvic injuries.

## Conclusion

Sacral fractures and PRIs are associated with high morbidity and mortality, making accurate and early diagnosis essential to enable appropriate management. We can be concluded that there are associations involving specific PRI subtypes and the most prevalent sacral fracture morphologies, particularly Tile B-Denis I and Tile C-Denis II. Recognizing these patterns may help guide diagnostic investigation and support clinical diagnosis.

## References

[1] Malgaigne J F Double vertical fractures of the pelvis. 1859 Clin Orthop Relat Res 2007 458 458 17 19 10.1097/BLO.0b013e31803defac 17473592

[2] Stahel P F Ziran N The pathophysiology of pelvic ring injuries: a review Patient Saf Surg 2024 18 01 16 10.1186/s13037-024-00396-x 38741186 PMC11092015

[3] Papakostidis C Giannoudis P V Pelvic ring injuries with haemodynamic instability: efficacy of pelvic packing, a systematic review Injury 2009 40 04 S53 S61 10.1016/j.injury.2009.10.037 19895954

[4] Perry K Mabrouk A Chauvin B J Pelvic Ring Injuries. [Updated 2024 Mar 2] Treasure Island, FL StatPearls Publishing 2025Jan-. Available from:https://www.ncbi.nlm.nih.gov/books/NBK544330/31335050

[5] Jakob D A Benjamin E R Cremonini C Demetriades D Management and outcomes of severe pelvic fractures in level I and II ACS verified trauma centers Am J Surg 2021 222 01 227 233 10.1016/j.amjsurg.2020.10.031 33131692

[6] Marmor M El Naga A N Barker J Matz J Stergiadou S Miclau T Management of Pelvic Ring Injury Patients With Hemodynamic Instability Front Surg 2020 7 588845 10.3389/fsurg.2020.588845 33282907 PMC7688898

[7] Valisena S Abboud A E Andereggen E Ansorge A Gamulin A High-energy blunt pelvic ring injury: dataset of patients and injury characteristics from a severely injured patients' registry Data Brief 2022 45 108740 10.1016/j.dib.2022.108740 36426001 PMC9679749

[8] De Ridder V A Whiting P S Balogh Z J Mir H R Schultz B J Routt M C Pelvic ring injuries: recent advances in diagnosis and treatment OTA Int 2023 6 03 e261 10.1097/OI9.0000000000000261 37533441 PMC10392441

[9] Aprato A Vergano L B Casiraghi A Consensus for management of sacral fractures: from the diagnosis to the treatment, with a focus on the role of decompression in sacral fractures J Orthop Traumatol 2023 24 01 46 10.1186/s10195-023-00726-2 37665518 PMC10477162

[10] Bellabarba C Stewart J D Ricci W M DiPasquale T G Bolhofner B R Midline sagittal sacral fractures in anterior-posterior compression pelvic ring injuries J Orthop Trauma 2003 17 01 32 37 10.1097/00005131-200301000-00005 12499965

[11] Denis F Davis S Comfort T Sacral fractures: an important problem. Retrospective analysis of 236 cases Clin Orthop Relat Res 1988 227 227 67 81 3338224

[12] Zhang Y Z Lu S Xu Y Q Shi J H Li Y B Feng Z L Application of navigation template to fixation of sacral fracture using three-dimensional reconstruction and reverse engineering technique Chin J Traumatol 2009 12 04 214 217 19635214

[13] Tile M Pelvic ring fractures: should they be fixed? J Bone Joint Surg Br 1988 70 01 1 12 10.1302/0301-620X.70B1.3276697 3276697

[14] Hsu J R Bear R R Dickson K F Open reduction internal fixation of displaced sacral fractures: technique and results Orthopedics 2010 33 10 730 10.3928/01477447-20100826-07 20954668

[15] Chen H W Liu G D Ou S Zhao G S Pan J Treatment of unstable sacral fractures with percutaneous reconstruction plate internal fixation Acta Cir Bras 2012 27 05 338 342 10.1590/s0102-86502012000500010 22666748

[16] Ruatti S Guillot S Brun J Which pelvic ring fractures are potentially lethal? Injury 2015 46 06 1059 1063 10.1016/j.injury.2015.01.041 25769199

[17] Chueire A G Carvalho G Santos AFd Pockel K PFilho,Fraturas do anel pélvico: estudo epidemiológico Acta Ortop Bras 2004 12 01 5 11 10.1590/S1413-78522004000100001

[18] Gänsslen A Pohlemann T Paul C Lobenhoffer P Tscherne H Epidemiology of pelvic ring injuries Injury 1996 27 01 S-A13 20 8762338

[19] Beckmann N Cai C CT characteristics of traumatic sacral fractures in association with pelvic ring injuries: correlation using the Young-Burgess classification system Emerg Radiol 2017 24 03 255 262 10.1007/s10140-016-1476-0 28004324

[20] Tötterman A Glott T Madsen J E Røise O Unstable sacral fractures: associated injuries and morbidity at 1 year Spine 2006 31 18 E628 E635 10.1097/01.brs.0000231961.03527.00 16915078

